# Perceptions and Behaviors of Healthcare Providers Regarding Sexuality in Cervical Cancer Care: A Qualitative Study

**DOI:** 10.3390/curroncol33050253

**Published:** 2026-04-28

**Authors:** Ida Ayu Md Vera Susiladewi, Yati Afiyanti, Allenidekania Allenidekania, Margaret Fitch

**Affiliations:** 1 Master of Nursing Program, Faculty of Nursing, Universitas Indonesia, Depok 16424, Indonesia; 2 Integrated Cancer Center, Bali Mandara General Hospital, Denpasar 80227, Bali, Indonesia; 3 Maternity Nursing Department, Faculty of Nursing, Universitas Indonesia, Depok 16424, Indonesia; 4 Oncology Nursing Study Program, Faculty of Nursing, Universitas Indonesia, Depok 16424, Indonesia; 5 Lawrence Bloomberg Faculty of Nursing, University of Toronto, Toronto, ON M5T1P8, Canada; marg.i.fitch@gmail.com

**Keywords:** sexuality, sexual health, health personnel, uterine cervical neoplasms

## Abstract

Sexuality is a fundamental aspect of human well-being, yet it is often disrupted in women diagnosed with cervical cancer. Although healthcare providers play a crucial role in supporting patients’ sexual health, the topic remains largely unaddressed in oncology settings. This study aimed to explore how healthcare providers perceive and respond to sexual health concerns among women with cervical cancer. A qualitative approach was conducted between August and November 2024 at Dharmais Cancer Hospital, Indonesia. Eighteen healthcare workers experienced in cancer care were selected through purposive sampling. Semi-structured interviews were conducted and analyzed thematically using Braun and Clarke’s six-phase framework with Nvivo 12 Plus software. The analysis revealed three main themes: (1) diverse perceptions of sexuality; (2) unmet sexual health needs; and (3) challenges to address sexual health. This study highlights that healthcare providers acknowledge the importance of addressing sexuality in cancer care but face numerous challenges that hinder open discussions in Indonesia. Integrating sexual health into routine oncology care and enhancing provider training are essential steps toward delivering more holistic and patient-centered care. These findings can improve sexual health care in cancer patients.

## Simple Summary

Cervical cancer deeply impacts a woman’s sexuality, yet this remains a neglected topic in many hospitals. We spoke with healthcare professionals at a leading cancer center in Indonesia to understand why these conversations are not happening. While staff recognize that sexual health is vital for a patient’s well-being, our study found that it is rarely discussed due to cultural barriers, personal discomfort, and a lack of formal training. These findings show that being a good healthcare provider means more than just treating the cancer; it requires supporting the whole person. To improve care, we must provide better training for medical teams and make sexual health a standard part of routine cancer support. This approach will help women with cervical cancer lead fuller, more confident lives after diagnosis.

## 1. Introduction

Cervical cancer is the fourth most common cancer among women globally, with more than 660,000 new cases and 350,000 deaths reported in 2022 [1]. In Indonesia, it is the second leading cause of cancer-related deaths among women, accounting for 36,964 new cases and 20,708 deaths in the same year [2]. Most women diagnosed with cervical cancer are in their reproductive and economically productive years, making the impact of the disease far-reaching, not only on their physical health but also on their psychosocial and sexual well-being.

Sexual dysfunction occurs in women with cervical cancer, particularly following therapeutic modalities such as radiotherapy and chemotherapy, which arise from multifactorial etiologies [3,4,5]. Sexual dysfunction can manifest as pain during intercourse (dyspareunia), vaginal dryness, loss of libido, and decreased emotional and physical intimacy, all of which can significantly affect a woman’s body image, emotional health, and quality of life [6,7]. These challenges often extend into personal relationships and can contribute to psychological distress, including anxiety, depression, and low self-esteem [3,8,9].

Despite extensive evidence highlighting the effects of cervical cancer on women’s sexuality, sexual health is still frequently overlooked in oncology care. In many settings, including Indonesia, discussions around sexuality remain culturally sensitive and are often avoided by both patients and healthcare providers [10,11]. Cultural factors, time constraints, and personal reservations among providers contribute to the invisibility of sexual health in clinical conversations [12,13].

In Indonesia, discussions of sexual health are often constrained by deep-seated cultural taboos and religious beliefs, which create a pervasive culture of silence [14,15]. Central to this is the Javanese concept of *malu* (shame) and the preservation of family honor, which often categorizes any discourse on sexuality as taboo or indecent. Furthermore, the strong influence of Islamic jurisprudence means that sexual matters are frequently viewed through the moral lens of *zina* (sinful carnal relations), leading many to fear that open discussion, even in a medical context, could be misinterpreted as promoting immorality. Gendered expectations also play a critical role; the traditional construct of *kodrat wanita* dictates that women should remain passive and modest regarding their bodies, often resulting in self-censorship to avoid being labeled as promiscuous. Additionally, sexuality education is frequently resisted as a perceived threat to *nilai-nilai ketimuran* (Eastern values) or a symptom of Western liberalization. These barriers limit both patients’ willingness to raise concerns and providers’ comfort in addressing them, as both parties navigate a landscape where sexual health is often sidelined to maintain social and religious standing [14,16]. Understanding these specific local perspectives is crucial for exploring how healthcare providers perceive and respond to sexual health issues in cervical cancer care, ensuring that interventions are both culturally sensitive and clinically effective.

Prior studies, across a variety of countries, highlight that awareness regarding the importance of sexual health is not matched by adequate fulfillment of the sexuality needs. Psychosexual shifts occurring after a cervical cancer diagnosis highlight the critical role of open dialogue in restructuring sexual identity and personal requirements in women. [17]. While many physicians believe sexual health is an essential component of care, clinical practice often falls short. Although some literature suggests a 50% inquiry rate [18], other surveys indicate that 31.6% of physicians rarely or never address it [10], with frequencies dropping further during treatment initiation (15.2%) and follow-up (33.1%) [19]. Nurses had positive attitudes towards discussing sexual health concerns with patients; however, they rarely did so [11,13]. Many providers report significant barriers to initiating sexual health conversations, primarily due to limited training, lack of practical training, time constraints, personal discomfort, unclear professional roles, fear of offending patients, and organizational factors [11,12,18]. However, most of these studies were conducted outside Indonesia.

Although the attitudes and beliefs of healthcare professionals regarding sexual health issues have been widely studied [9], perceptions and behaviors in addressing sexual health concerns remain limited in Indonesia. Empirical studies examining sexual health services among Indonesian oncologists, nurses, or midwives are scarce. There is a notable scarcity of recent studies exploring how healthcare providers address patients’ sexual concerns in Indonesia. In the absence of clear national guidelines and structured training, sexual health care is frequently fragmented, with responsibilities implicitly shifted across professional roles. These findings highlight a significant gap in the Indonesian context and underscore the need for further investigation into culturally appropriate strategies to support sexual health in oncology care.

Accordingly, the central research question guiding this study is: How do healthcare professionals in Indonesia perceive and respond to sexual health issues in the care of patients with cervical cancer? The study seeks to provide a deeper understanding of how healthcare providers in this country perceive, experience, and manage sexual health issues and to identify the challenges associated with their roles in delivering comprehensive sexual care. The study will address gaps in the literature and contribute evidence from the context of Indonesia.

## 2. Materials and Methods

### 2.1. Study Design and Setting

A qualitative descriptive design was used for this study, as it is well-suited for investigating real-world clinical practices and obtaining rich, experience-based insights in natural settings. This study was underpinned by a descriptive thematic analysis approach, which enabled the systematic identification and interpretation of patterns in participants’ narratives to generate a comprehensive understanding of their perspectives.

The study followed the Consolidated Criteria for Reporting Qualitative Research (COREQ-32) checklist [20], ensuring transparency across three domains: research team and reflexivity, study design, and data analysis. The study was conducted at Dharmais Cancer Hospital in Indonesia, a leading tertiary referral hospital specializing in oncology care from March to November 2024.

### 2.2. Research Team and Reflexivity

Interviews were conducted by the first author (IAMVS). She is a master’s student in oncology nursing who has received formal training in qualitative interviewing. No professional or hierarchical relationship existed between the interviewer and the participants, and the interviewer was not affiliated with the study site. This helped minimize power imbalances and encourage honest, open dialogue. Supervision was provided by YA, a professor of maternity nursing with extensive experience in qualitative research, who supported methodological planning and oversight throughout the study. AA is a senior lecturer who attended each interview as note-taker and observer and contributed to reflexive discussions and thematic development during data analysis. Reflexive journals and field notes were maintained by the research team to support self-awareness and reduce the influence of potential biases.

### 2.3. Study Participants and Recruitment

A purposive sampling with maximum variation was used to ensure a diverse set of perspectives. Inclusion criteria were (1) active employment as a healthcare provider; (2) a minimum of two years of experience in cervical cancer care; (3) current assignment in either inpatient or outpatient settings; and (4) willingness to participate voluntarily. Recruitment and interviews were conducted between August and November 2024 in private areas within a national cancer center to ensure confidentiality. Recruitment was facilitated by department supervisors and continued until data saturation was reached.

Participants were informed about the study objectives and procedures. They had no prior relationship with the researcher and were not aware of the researcher’s personal goals or reasons for conducting the study. No individual invited to participate in the study declined to take part.

### 2.4. Data Collection

Participants were informed about the study objectives, procedures, and confidentiality measures. Written informed consent was obtained for both participation and audio recording. Semi-structured interviews were conducted face to face in Bahasa Indonesia using an interview guide developed from a comprehensive literature review and insights from clinical oncology practice (Table 1). The interviews lasted between 45 and 75 min.

Topics explored included participants’ understanding of the concept of sexuality, discussing sexual health with patients, perceived challenges in approaching the issues of sexual needs, and their suggestions to solve sexual issues. The guide included open-ended questions and follow-up prompts to facilitate in-depth exploration of participants’ perspectives. The interview questions were pilot tested with two oncology nurses to evaluate clarity, cultural appropriateness, and feasibility. Feedback from the pilot interviews was used to refine question wording and sequencing. Data from the pilot interviews were not included in the main analysis.

Interviews were held in quiet, private hospital rooms with the participants’ consent. No repeat interviews were conducted; each participant was interviewed once. Only the participant and the researchers were present during an interview session. After each session, participants completed a short questionnaire capturing demographic and professional information (Table 2). Audio recordings were transcribed verbatim in Bahasa Indonesia, then verified against the recordings to ensure accuracy. Transcripts were returned to participants for their review, comments, and corrections to ensure the credibility of the data.

### 2.5. Rigor and Trustworthiness

To ensure the study’s rigor, Lincoln and Guba’s four criteria for trustworthiness were applied [21,22]: credibility was strengthened through triangulation (researchers, professions, and sources), prolonged engagement, and member checking to verify accuracy of findings. Participants provided feedback on the findings through member checking to validate the accuracy and credibility of the interpretations. Dependability achieved by maintaining a clear audit trail of all methodological and analytical decisions, along with detailed field notes. Confirmability ensured through peer debriefing, reflexive journaling, and collaborative discussions to minimize bias in data interpretation. Transferability was facilitated by providing thick descriptions of the study setting, participants, and data collection process to allow readers to judge relevance to other contexts.

### 2.6. Data Analysis

Thematic analysis was conducted following Braun and Clarke’s six-phase framework: (1) familiarization with the data, (2) generating initial codes, (3) searching for themes, (4) reviewing themes, (5) defining and naming themes, and (6) producing the report [23,24]. An inductive approach was used, allowing patterns and themes to emerge from the data without imposing a pre-existing framework.

Transcripts were reviewed repeatedly to ensure data immersion. Initial coding was conducted independently by IAMVS and AA, followed by team-based comparison and refinement of the coding structure through collaborative discussions with YA. Discrepancies were resolved through consensus. NVivo 12 Plus Software (Version 12.6.0.959; QSR International, Burlington, MA, USA) was used to manage data and support coding organization. Trustworthiness during analysis was reinforced through peer debriefing, multidisciplinary triangulation, member checking, and consistent documentation of analytic decisions in an audit trail.

Data saturation was achieved during the process of in-depth interviews with healthcare professionals involved in cervical cancer care. Initially, interviews were conducted with a diverse group of healthcare providers to capture a wide range of perceptions and behaviors related to sexual health care. To ensure rigor, the researchers conducted three additional interviews to confirm that no new themes emerged, thereby strengthening confidence that saturation had been reached.

During the preparation of this manuscript, the authors used the generative AI features of Grammarly (Grammarly Inc., San Francisco, CA, USA; https://www.grammarly.com) to assist in drafting, restructuring, and refining the clarity of the text. Specifically, the tool was utilized to improve the flow of arguments and ensure linguistic precision. The authors take full responsibility for the originality, validity, and integrity of the content, including the portions modified by the AI tool. All AI-generated suggestions were critically reviewed and edited by the authors to ensure they accurately represent the study’s data and findings.

### 2.7. Ethical Considerations

Ethical approval was granted by the Ethics Committee of Dharmais Cancer Hospital, document number DP.04.03/11.5/138/2024. Participant autonomy and privacy were the central ethical considerations of this study. Informed consent was secured by the first author, with a guarantee of total anonymity, as no identifying data were recorded. Furthermore, data security protocols were established to ensure that collected information remained accessible only to the research team.

## 3. Results

A total of eighteen healthcare providers, selected purposively to ensure a diverse representation of professional roles, consisting of five nurses, four doctors, two midwives, two clinical psychologists, two nutritionists, two physiotherapists, and a clinical pharmacist involved in the care of patients with cervical cancer. Participants ranged in age from 27 to 58 years and had between 4 and 34 years of experience in the healthcare field. All participants had a minimum of two years of experience caring for women with cervical cancer in either inpatient or outpatient oncology settings (Table 2). Based on the thematic analysis, several themes and subthemes emerged regarding the providers’ experiences and perspectives, which are systematically summarized in Table 3.

### 3.1. Theme 1: Diverse Perceptions of Sexuality

This theme describes the diverse perspectives of healthcare professionals regarding the concept of sexuality, especially in the context of cervical cancer care. It comprises two subthemes: (i) sexuality as a part of human life, and (ii) sexuality in patient care.

#### 3.1.1. Sexuality as a Part of Human Life

Participants consistently viewed sexuality as a fundamental human need. However, the definition of sexuality varied across professions, genders, and personal experiences. While some viewed sexuality in a broader psychosocial and emotional context, others held a more physical and biological perspective.

The majority emphasized that sexuality was not solely about sexual intercourse but included emotional connection, identity, and relational harmony.

*“...for me, sexuality includes physical, emotional, and psychological aspects, and even social, within the context of relationships—both internal with oneself and external with a spouse...”*

(P3, Physician)

One male physician associated sexuality with reproduction, while two unmarried female psychologists and a physiotherapist emphasized mutual romantic fulfillment within a partnership. Some participants also linked sexuality to religious and cultural values, describing it as sacred.

#### 3.1.2. Sexuality in Patient Care

Participants acknowledged the relevance of sexuality in cervical cancer care but admitted it was rarely addressed in practice. Although patients occasionally raised sexual concerns, healthcare providers often felt unprepared or constrained by clinical priorities. Discussions about sexuality were generally deprioritized, undocumented, and lacked follow-up.

*“…we ignore it because the focus is on chemotherapy... if it’s not in the notes, we don’t address it...”*

(P2, Nurse)

Roles in addressing sexual health were unclear. While physicians were often seen as primarily responsible, nurses were usually the first to hear about such concerns. Some providers believed that managing sexual problems required collaboration, while others deferred to gynecologists or psychologists.

*“…if it’s about emotions, we refer to psychologists; if it’s about surgery, we refer to the surgeon...”*

(P13, Physiotherapist)

A few participants reported educating patients on maintaining sexual activity after treatment to prevent complications such as vaginal adhesions. However, most admitted that no structured intervention or coordination system was in place to manage patients’ sexual concerns.

*“…we tell them intercourse is necessary after radiation to prevent narrowing of the vagina...”*

(P6, Nurse)

Overall, the perspectives reflect a lack of consistent, integrated approaches to sexuality in cervical cancer care despite participants’ recognition of its importance.

### 3.2. Theme 2: Unmet Sexual Health Needs

This theme captures the participants’ views on the unaddressed sexual health needs of patients with cervical cancer, resulting in significant gaps in care. Although participants recognized these concerns, sexual issues were often left unmanaged during the care process, leading to unmet patient needs. Two key subthemes were identified: patient-related factors and provider-related factors.

#### 3.2.1. Patient-Related Factors

Participants described how patients with cervical cancer frequently experienced relational and sexual concerns that were not adequately explored or managed by healthcare providers. Many patients expressed anxiety about their partner’s loyalty, fears of physical changes, and uncertainty about sexual activity but received limited support.

*“...they often ask, ‘If I’m like this, will my husband still want to be with me? Or will he start looking for someone else?’ but that’s rarely discussed. Not all nurses are capable of exploring sexual issues...”*

(P12, Nurse)

*“...some patients ask, ‘Will my sex life be affected?’ Honestly, it’s hard to answer that, the doctors know better...”*

(P13, Physiotherapist)

Patients also struggled with the physical effects of treatment, such as vaginal stenosis, and were often unaware of ways to prevent complications due to a lack of early education.

*“...she told me, ‘They examined me and said my vagina is closed.’ She felt incomplete as a woman. That could have been prevented with better education early on...”*

(P12, Nurse)

While some providers became aware of these needs through standardized questionnaires, they did not follow up unless the patient initiated the discussion.

*“...we notice it from EORTC QLQ-CX24 [a validated cervical cancer specific module asessing symptom distress], like when a young patient reports no sexual activity. But unless she brings it up, we don’t address it...”*

(P17, Doctor)

Participants noted that patients often lacked access to accurate information and did not know whom to approach. In some cases, patients end up asking unrelated staff, such as pharmacists.

*“...maybe her biological needs were overwhelming, and her husband was also not fulfilled. She didn’t know whom to ask, so she tried asking the pharmacist when collecting her meds...”*

(P11, Pharmacist)

Furthermore, patients expressed a desire for their concerns to be acknowledged and addressed, yet gender dynamics and limited access to female doctors hindered communication.

*“...patients often feel more comfortable talking to female doctors, but most of our OB/GYNs are male, except one...”*

(P17, Doctor)

#### 3.2.2. Provider-Related Factors

Participants expressed a desire to meet patients’ sexual health needs but felt constrained by multiple barriers. Time limitations was one of the most frequently cited issues. Short consultations and large patient loads made it difficult to provide thorough information.

*“...in the outpatient clinic, there’s just not enough time. If patients ask, we try to explain, but not everyone gets the information they need...”*

(P14, Midwife)

*“...ideally, there should be dedicated education about sexology, but honestly, there’s no specific time allocated for it...”*

(P2, Nurse)

Lack of motivation was another key barrier. Although nurses understood that sexuality is a basic human need, they admitted to avoiding the topic due to discomfort, lack of clear guidelines, and perceived difficulty in assessing and evaluating sexual concerns.

*“...nurses know very well that sexuality is a basic human need, but to motivate them to explore it? Sometimes they just don’t feel like asking...”*

(P12, Nurse)

*“...assessing sexuality is hard, the interventions are complex, and evaluation isn’t easy either. That’s why we often set it aside...”*

(P6, Nurse)

Participants also felt unsupported by related parties. Although patients’ partners were considered essential in managing sexual issues, providers noted that most patients attended consultations alone. Moreover, existing support systems such as cancer patient navigators had not yet been widely recognized or utilized for sexual health concerns.

*“...many patients come alone, without their partners, so we can’t provide couple-based education...”*

(P13, Physiotherapist)

### 3.3. Theme 3: Challenges to Address Sexual Health

This theme highlights the various challenges faced by healthcare providers in addressing sexual issues in cancer care, particularly for patients with cervical cancer. Participants admitted that they often felt unprepared and lacked the necessary competence to manage sexual health concerns. Many stated they had never received formal training in sexuality-related communication or counseling and expressed a need for regular, structured education to build their knowledge and skills.

*“...when it comes to psychosocial, emotional, and also sexual issues, we struggle to manage them because they’re outside our area of expertise...”*

(P3, General Practitioner)

*“...we need additional knowledge and training because educating and communicating are skills. It’s not just about the knowledge...”*

(P6, Nurse)

Healthcare professionals also pointed out the absence of standard protocols and integrated education materials to guide sexual health discussions in routine care. Several participants emphasized the importance of initiating sexual health education even when patients do not raise the topic themselves.

*“...honestly, I feel that I’m not capable of evaluating that [sexual problem]...”*

(P18, Clinical Psychologist)

*“...to provide holistic care, we need a guideline on what should be included in the education...”*

(P8, General Practitioner)

Environmental constraints, such as a lack of private consultation spaces, were also reported as barriers to discussing sensitive topics.

*“...the rooms aren’t very private. In rehabilitation ward, we only use partitions, and the sliding doors don’t fully close...”*

(P17, Nurse)

To address time constraints and ensure comprehensive care, participants suggested incorporating self-administered screening tools as part of the standard procedure.

*“...maybe a self-administered questionnaire for screening is needed. We should also provide more education, not only physical but about sexuality too...”*

(P3, General Practitioner)

Overall, the participants expressed a strong desire for institutional support in the form of training, tools, and infrastructure to help them manage patients’ sexual concerns more effectively and holistically.

## 4. Discussion

This study provides an in-depth understanding of how healthcare providers in Indonesia perceive and address sexual health in the care of women with cervical cancer. The findings highlight that, although participants acknowledged the importance of sexuality as part of holistic care, addressing it in practice remains limited due to intersecting personal, sociocultural, and institutional factors.

Consistent with previous research conducted in other countries, the study found that healthcare uncertainty is due to misperceptions regarding the significance of the topic, a fear of causing offense, and a lack of specific knowledge to guide treatment during clinical conversations about sexuality [25,26,27,28,29,30]. These narratives reflect relational avoidance and boundary ambiguity, where clinicians’ fear of causing offense or overstepping professional roles leads to the systematic postponement of essential sexual health discussions. Consistent with recent literature, this psychological impasse results in clinical avoidance, prioritizing immediate social comfort over holistic patient care [30,31,32].

Cultural values surrounding modesty, gender, and privacy played a significant role in shaping providers’ behaviors. These influences align with broader societal norms in Indonesia, where open conversations about sexuality are often viewed as inappropriate, particularly in healthcare interactions. Participants’ hesitations reflect socio-cultural scripts and professional habitus, where clinical behavior is moderated by community expectations and perceived taboos. Consistent with recent scholarship on high-context cultural norms, these findings underscore how professional identity often prioritizes the preservation of modesty over clinical transparency, reinforcing a cycle of silence in sexual health care [33,34,35,36].

While clinical avoidance is a documented global phenomenon [10,11,18], the drivers in Indonesia are uniquely shaped by the intersection of religious morality and cultural modesty. Unlike Western contexts, where barriers are often structural (e.g., time or privacy), our findings suggest a psychological impasse deeply rooted in the fear of moral judgment. This aligns with other high-context and Muslim-majority cultures, where preserving social harmony and adherence to collective moral standards often take precedence over individual health disclosures [33].

The absence of institutional support further complicated these dynamics. Participants identified systemic issues such as the lack of sexual health guidelines, limited access to training, and time constraints in busy oncology units. These results align with contemporary oncology literature, which identifies systemic institutional neglect as a primary driver in the persistent marginalization of sexual health services within cancer care. Recent studies suggest that when organizational frameworks fail to prioritize psychosexual well-being, it creates a structural barrier that transcends individual provider intent, effectively relegating sexual health to a peripheral concern rather than a core component of comprehensive survivorship care [27,37].

Without adequate policies, training infrastructure, or role clarity, even motivated providers felt unable to initiate sensitive conversations confidently. To address this gap, Indonesia could benefit from adapting established international frameworks tailored to the local socio-religious context. For instance, the PLISSIT model (Permission, Limited Information, Specific Suggestions, and Intensive Therapy) provides a stepped approach that allows providers to offer ‘Permission’ to discuss sexuality, effectively lowering the barrier of malu (shame). Additionally, the ASCO (American Society of Clinical Oncology) guidelines and the NCCN (National Comprehensive Cancer Network) survivorship protocols offer structured algorithms that could provide the much-needed role clarity and standardized scripts for Indonesian clinicians, ensuring that sexual health is addressed as a routine medical side effect rather than a sensitive personal issue.

Contemporary research increasingly emphasizes a shift toward proactive clinical frameworks over reactive care. While participants demonstrated significant personal motivation to enhance their sexual health competencies, the recent literature suggests that willingness must be harnessed through systemic integration rather than individual effort alone. To bridge this gap, participants proposed adopting standardized clinical protocols and fostering interdisciplinary synergy, particularly by embedding psychologists and specialized counselors into the core oncology team. This aligns with current trends favoring anticipatory guidance, which advocates for the normalization of sexual health by introducing it as a routine component of the patient’s initial care trajectory rather than a late-stage intervention [36,38].

### 4.1. Significance

This study reinforces the concept of multidimensional unmet needs by showing that clinical awareness is often hindered by structural and interpersonal barriers, shifting the focus from individual provider willingness to institutional accountability. It argues that meaningful progress in sexual health requires a systemic overhaul, encompassing policy reform, integrated curricula, and supportive organizational cultures, to transform provider recognition into actionable, standard care [39,40,41].

This study contributes to nursing science by deepening the understanding of how cultural, personal, and institutional factors intersect to shape healthcare providers’ practices in addressing sexual health among women with cervical cancer. The multidisciplinary findings necessitate role-specific actions: doctors and midwives should initiate proactive screening using tools like the EORTC QLQ-CX24 to normalize the topic; nurses must integrate sexual health into routine holistic care to reduce patient malu (shame); and clinical psychologists should address the deep-seated psychosexual impact of cultural stigmas. Furthermore, physiotherapists play a key role in managing physical barriers (e.g., pelvic floor health), while nutritionists and clinical pharmacists must provide anticipatory guidance on how treatment side effects and dietary changes may indirectly impact sexual vitality. Collectively, these efforts require hospital management to provide the structural support and privacy necessary to transform individual intent into standard clinical practice.

The findings emphasize that nurses, as key providers of holistic care, play a central role in recognizing sexuality as an integral component of patient well-being. By identifying the barriers of discomfort, sociocultural taboos, and lack of institutional support, this study highlights the need for culturally sensitive communication training, policy development, and structured protocols in oncology nursing.

### 4.2. Strengths and Limitations

This study used the COREQ framework to guide methodological rigor, including researcher reflexivity, triangulation of perspectives, and thick description of participant experiences. The multidisciplinary sample also provided a comprehensive view of sexual care challenges across healthcare roles, enhancing the richness and transferability of the findings. The reflexive approach adopted by the research team. The lead interviewer had no clinical or hierarchical relationship with participants and was not affiliated with the institution, which helped reduce power imbalances. Reflexive journals, team debriefings and member checking also contributed to the trustworthiness of the findings.

However, some limitations must be acknowledged. First, the findings reflect the cultural and institutional context of a single national cancer center in Indonesia, which may limit generalizability to other settings. Second, due to the sensitivity of the topic, some participants may have hesitated to share openly, potentially restricting the depth of certain narratives. The risk of participant hesitation due to the sensitive nature of the topic was minimized by the researcher’s month-long engagement and observation prior to the study, which fostered a sense of familiarity and safety.

Another limitation relates to the researcher’s positionality and limited experience in sexuality-focused research. While this may have influenced data interpretation, strategies such as bracketing, supervision, and cross-checking of translations were used to minimize bias and enhance analytic neutrality.

Despite these limitations, the study offers valuable insights into the emotional, cultural, and systemic dimensions of sexual health in oncology. It highlights key areas for intervention and lays the groundwork for future research and training programs aimed at improving sexual care for women with cervical cancer.

## 5. Conclusions

This study highlights the complex and multifaceted nature of addressing sexual health within cervical cancer care from a multidisciplinary healthcare perspective. Although healthcare professionals recognized sexuality as a vital component of holistic, person-centered care, their ability to support patients was often constrained by a combination of personal discomfort, cultural taboos, limited training, and a lack of institutional support.

The absence of clear clinical guidelines, inadequate education, and minimal organizational emphasis on sexual health contributed to the ongoing neglect of patients’ sexual concerns. These findings reveal how systemic barriers reinforce silence around sexuality, even when providers are aware of its relevance to patients’ quality of life and psychological well-being.

A key contribution of this study is the identification of persistent unmet needs related to sexual health in oncology care. This signals an urgent need for systemic change, beyond individual awareness, through the implementation of supportive policies, training programs, and communication frameworks that enable healthcare providers to address sexual concerns confidently and compassionately.

By exploring provider perspectives within their cultural and professional contexts, this study offers valuable insights into the realities of sexual health communication in oncology settings. The findings provide a foundation for developing practical and culturally responsive strategies that promote more equitable, comprehensive care for women living with cervical cancer.

## Figures and Tables

**Table 1:** Semi-structured interview guide.

Number	Domains	Question
1	Conceptualization	What is your understanding of sexual health aspects and their specific domains?
2	Clinical Standard	In your view, what characterizes a healthy sexual aspect within a clinical context?
3	Practice and Experience	Have patients with cervical cancer expressed sexual concerns? How did you manage these situations, and to whom did you refer if necessary?
4	Collaboration	How are patients’ sexual problems discussed and managed within your multidisciplinary team?
5	Facilitators	What factors (environmental, patient-related, or organizational) facilitate these discussions?
6	Barriers	What factors (personal, organizational, or cultural) prevent you from assessing or intervening in patients’ sexual concerns?

**Table 2:** Participants’ Characteristics.

Participants	Age	Gender	Profession	Experience (Years)	Education Level	Marital Status
P1	43	Female	Nurse	21	Bachelor’s degree	Married
P2	50	Female	Nurse	31	Bachelor’s degree	Married
P3	38	Female	Doctor	7	Master’s/Specialist	Not married
P4	27	Female	Nutritionist	5	Diploma	Not married
P5	40	Female	Midwife	19	Diploma	Married
P6	44	Female	Nurse	21	Bachelor’s degree	Married
P7	41	Male	Doctor	6	Master’s/Specialist	Married
P8	30	Female	Doctor	4	Bachelor’s degree	Not married
P9	43	Female	Nurse	23	Bachelor’s degree	Married
P10	28	Female	Nutritionist	6	Bachelor’s degree	Not married
P11	30	Female	Clinical pharmacist	5	Bachelor’s degree	Married
P12	47	Female	Nurse	25	Master’s/Specialist	Married
P13	58	Male	Physiotherapist	34	Bachelor’s degree	Married
P14	32	Female	Midwife	10	Bachelor’s degree	Married
P15	32	Female	Physiotherapist	11	Bachelor’s degree	Not married
P16	32	Female	Clinical psychologist	6	Master’s/Specialist	Not married
P17	37	Female	Doctor	6	Master’s/Specialist	Married
P18	32	Female	Clinical psychologist	4	Master’s/Specialist	Not married

**Table 3:** Themes and subthemes.

Themes	Subthemes
Diverse perceptions of sexuality	As a part of human life Sexuality in patient care
Unmet needs in sexual health	Patient-related factors Healthcare provider-related factors
Challenges to address sexual health	-

## Data Availability

The data underlying the study’s findings are not publicly accessible due to participant privacy and confidentiality and ethical constraints.
